# Golay Complementary Waveforms in Reed–Müller Sequences for Radar Detection of Nonzero Doppler Targets

**DOI:** 10.3390/s18010192

**Published:** 2018-01-11

**Authors:** Jiahua Zhu, Xuezhi Wang, Xiaotao Huang, Sofia Suvorova, Bill Moran

**Affiliations:** 1 College of Electronic Science, National University of Defense Technology, Changsha 410073, China; xthuang@nudt.edu.cn; 2 School of Engineering, RMIT University, Melbourne, VIC 3000, Australia; xuezhi.wang@rmit.edu.au (X.W.); sofia.suvorova@unimelb.edu.au (S.S.); wmoran@unimelb.edu.au (B.M.); 3 Department of Electrical and Electronic Engineering, The University of Melbourne, Melbourne, VIC 3010, Australia; 4 Collaborative Innovation Center of Information Sensing and Understanding, Changsha 410073, China

**Keywords:** Golay complementary waveforms, Reed–Müller sequences, sidelobes suppression, multiple nonzero Doppler targets, detection

## Abstract

Golay complementary waveforms can, in theory, yield radar returns of high range resolution with essentially zero sidelobes. In practice, when deployed conventionally, while high signal-to-noise ratios can be achieved for static target detection, significant range sidelobes are generated by target returns of nonzero Doppler causing unreliable detection. We consider signal processing techniques using Golay complementary waveforms to improve radar detection performance in scenarios involving multiple nonzero Doppler targets. A signal processing procedure based on an existing, so called, Binomial Design algorithm that alters the transmission order of Golay complementary waveforms and weights the returns is proposed in an attempt to achieve an enhanced illumination performance. The procedure applies one of three proposed waveform transmission ordering algorithms, followed by a pointwise nonlinear processor combining the outputs of the Binomial Design algorithm and one of the ordering algorithms. The computational complexity of the Binomial Design algorithm and the three ordering algorithms are compared, and a statistical analysis of the performance of the pointwise nonlinear processing is given. Estimation of the areas in the Delay–Doppler map occupied by significant range sidelobes for given targets are also discussed. Numerical simulations for the comparison of the performances of the Binomial Design algorithm and the three ordering algorithms are presented for both fixed and randomized target locations. The simulation results demonstrate that the proposed signal processing procedure has a better detection performance in terms of lower sidelobes and higher Doppler resolution in the presence of multiple nonzero Doppler targets compared to existing methods.

## 1. Introduction

Advanced signal processing techniques have long been used for radar detection to improve receiver signal-to-noise ratio (SNR) and target return range resolution. For example, by use of matched filtering between the received signal and the transmitted waveform, the amplitude of output will be accumulated at the target return delay bin [1], which is close to the ideal impulse response. Waveforms that can generate impulse-like autocorrelation functions are of great interest in target detection and localization. The linear frequency modulation (LFM)/chirp waveform is one of the most common of such waveforms in practice. Its simplicity of generation and high time-bandwidth product make the LFM waveform an obvious choice [2]. Nevertheless, such LFM waveforms are well known to conflate range and Doppler as indicated by their ambiguity function (AF), and their target detection performance is limited by associated sidelobe issues. Researchers have addressed these problems with LFM waveforms in the literature and some relatively recent representative works are briefly described below. Santra et al. [3] use optimal matched illumination constant envelope waveforms obtained via phase retrieval techniques to demonstrate superior resolution characteristics compared with classical LFM waveforms employing optimal pulse compression. A tracking algorithm using a particle filter that selects and configures LFM or a nonlinear frequency modulation waveform in the transmitter is proposed by Sira et al. [4], for better detection of target and estimation of its current state. Rasool et al. proposed a V-chirp waveform in [5], inspired by the echo-location method of bats, for a significant enhancement of range and Doppler resolution, compared to LFM waveforms, with only a slight loss of detection probability, and Zhu et al. [6] extends the waveform scheme to the double V-chirp waveform, to suppress false targets and further enhance resolution in multiple target scenarios. Other waveform design schemes in place of LFM waveforms, such as orthogonal frequency division multiplexing (OFDM) and OFDM-chirp waveforms [7,8,9,10], or multiple-input multiple-output (MIMO) radar design [11,12,13,14], and waveform libraries scheduling [15,16,17] can also be deployed to improve detection and resolution performance of radar, but more complex waveform styles and hardware structures are required for these approaches.

In addition to the approaches described above, phase coded waveforms described in [18] are commonly used to achieve impulse-like autocorrelations. These use a biphase or polyphase unimodular sequence to phase code a long transmission pulse, so that the autocorrelation function of the coded waveform is controlled by the autocorrelation function of the unimodular sequence, which provides tighter control of the autocorrelation (though possibly requiring more transmission time) than conventional waveform schemes, and can be achieved easily in modern hardware systems. Frank codes [19], Barker codes [20] and other kinds of polyphase sequences [21,22] have been widely used in recent decades, though they are incapable of yielding an impulse aperiodic autocorrelation through the transmission of a single phase coding sequence [23]. The idea of using complementary sets of phase coding waveforms has been proposed many times. Predominantly Golay complementary waveforms [24], or their variants, have been suggested. These special designed waveforms have the property that the sum of their autocorrelation functions vanishes at all nonzero lags; thus, pairs of Golay complementary waveforms achieve an impulse autocorrelation output without any sidelobes, at least in theory. Numerous research publications have promoted this concept [23,25,26,27,28,29,30], but, as is widely understood, standard Golay complementary waveforms suffer two disadvantages; firstly, they have to be transmitted in pairs and those pairs have to completely separated on their return to the receiver and, secondly, they are sensitive to the mismatch of Doppler. Significant range sidelobes occur in nonzero Doppler lines in the AF.

To solve this problem, several schemes have been proposed by researchers. Calderbank et al. [31] and Pezeshki et al. [23] observe that the transmission order of Golay complementary waveforms significantly influences the range sidelobes level. They find that, by ordering the waveforms suitably, the range sidelobes in the AF near zero-Doppler can be significanltly suppressed. Based on that work, Dang et al. [32,33] proposed a Binomial Design (BD) algorithm that, in addition to re-ordering the waveforms, applies weights to the matched filtering. This significantly expands the range sidelobe blanking area around zero-Doppler in the AF and reduces target detection uncertainty. In a parallel direction, Suvorova et al. [34,35] proposed an algorithm for determining the transmission order of Golay complementary waveforms using Reed–Müller codes so that the range sidelobes of AF in the region centered at a given (nonzero) target Doppler are minimized. On the other hand, Levanon et al. [36] described the shortcoming of using Golay complementary waveforms and suggested methods to mitigate some of them. They indicated that a poor performance can be observed in the AF if both the transmission order algorithm and receiving weighting approach are applied rather than use only one of them.

While these methods provide significant improvements, further improvements are desirable and possible. In Dang’s work, the improvement of SNR is only for targets with low Doppler. In the presence of multiple targets with nonzero Doppler shifts, weak targets continue to be submerged in the sidelobes from strong targets. In addition, the expansion of the range sidelobe blanking area by the Binomial Design algorithm is at the expense of reduced Doppler resolution. In a similar way, the results of Suvorova et al., while addressing this poor detection issue for a specific nonzero Doppler target, only apply to a single Doppler value.

We propose a new signal processing procedure to enhance radar illumination performance of multiple nonzero Doppler targets by applying a combination of the Reed–Müller code method of Suvorova et al. and the Binomial Design algorithm of Dang et al. via a pointwise nonlinear procedure. We assume that a radar tracking system is available to provide prior information of target locations required in the proposed signal processing procedure. Numerical simulation results are presented to demonstrate the effectiveness of the proposed procedure. The major contributions of this paper are (1) to propose a novel signal processing procedure for the enhancement of detection and resolution performance; (2) to provide three transmission order design algorithms for Golay complementary waveforms under this procedure; and (3) to justify the effectiveness of the pointwise nonlinear processor with respect to target detection in theoretical calculations and in Monte Carlo simulation.

The paper is organized as follows. In Section 2, we briefly introduce Golay complementary waveforms and Reed–Müller codes as needed for this work. In Section 3, we first describe the Binomial Design algorithm and present three ways to order the Golay complementary waveforms using Reed–Müller sequences. A pointwise minimization procedure is then applied at the receiver between one of three orderings of the waveforms and the Binomial Design algorithm to obtain the final Delay–Doppler map. In Section 4, the computational complexities of all algorithms are analyzed and the use of pointwise minimization is explained in terms of acceptable performance, in order to provide a practical justification of the proposed algorithms. We also estimate regions in the Delay–Doppler map in which there are significant range sidelobes, induced by the presence of targets, for each of the three ordering algorithms, in an attempt to improve separation of nonzero Doppler targets. Numerical simulations for both fixed and randomized target detection scenarios are presented in Section 5, followed by the conclusions in Section 6.

## 2. Golay Complementary Waveforms in Reed–Müller Sequences

A pair of Golay complementary waveforms as considered in this work consists of two length *L* unimodular binary sequences x(l) and y(l), also referred to as a Golay complementary pair (details on the generation of Golay complementary pairs can be found in [24]). The chip interval of ±1 in a Golay complementary pair is fixed here as $T_{c}$, so that the total time duration of each sequence in the pair is $LT_{c}$. The key defining feature of a Golay complementary pair is that, for k=−(L−1),…,(L−1), the autocorrelation satisfies

(1) $$C_{x}\left(k\right)+C_{y}\left(k\right)=2L\delta \left(k\right),$$

where $C_{x}\left(k\right)$ and $C_{y}\left(k\right)$ are the autocorrelations of x(l) and y(l) at lag *k*, respectively, and δ(k) is the Kronecker delta function. The width of the impulse interval of δ(k) is ${2T}_{c}$, and this provides the range resolution of Golay complementary waveforms after modulation on the carrier. Modulation of a baseband pulse Ω(t) on each chip interval of the Golay complementary pair, the two sequences become the following time domain waveforms:

(2) $$\left\{\begin{array}{c} x\left(t\right)=\sum_{l=0}^{L-1}x\left(l\right)\Omega \left(t-lT_{c}\right),\\ y\left(t\right)=\sum_{l=0}^{L-1}y\left(l\right)\Omega \left(t-lT_{c}\right),\\ \int_{-T_{c}/2}^{T_{c}/2}|\Omega \left(t\right){|}^{2}dt=1. \end{array}\right.$$

Ideally, Ω(t) is a rectangular pulse, and this has been used in our simulations for simplicity, but, in a real system, the rectangle pulse would typically be replaced by another pulse shape, such as a raised cosine or Gaussian pulse to reduce the bandwidth requirement. A pulse train of Golay complementary waveforms is specified by a sequence pair (P,Q), which determines the transmission order and the weights on the received pulses; that is, the binary sequence $P={\left\{p\left(n\right)\right\}}_{n=0}^{N-1}$ determines whether x(t) or y(t) is transmitted at pulse (n+1), and the real sequence $Q={\left\{q\left(n\right)\right\}}_{n=0}^{N-1}$ provides the weights on each pulse. The transmitted signal is then expressed as

(3) $$z_{P}\left(t\right)=\sum_{n=0}^{N-1}p(n)x(t-nT)+(1-p(n))y(t-nT),$$

where *T* is the pulse repetition interval (PRI), and *N* is the number of pulse. For example, the (n+1)th pulse in $z_{P}\left(t\right)$ is x(t), if p(n)=1 and is y(t), if p(n)=0. The alternating sequence P={1,0,1,0,…} is called the standard transmission order for Golay complementary waveforms. Bearing in mind the weight sequence *Q*, the match filtering pulse train at the receiver is

(4) $$z_{Q}\left(t\right)=\sum_{n=0}^{N-1}q(n)[p(n)x(t-nT)+(1-p(n))y(t-nT)].$$

Usually, *Q* is an all 1 sequence, but, in some cases (e.g., the Binomial Design algorithm described in the next section), it may have values other than 1.

According to the definition in [1], the AF of a Golay complementary pair can be written as

(5) $$\chi_{PQ}\left(t,F_{D}\right)=\int_{-\infty }^{+\infty }z_{P}\left(s\right)\exp\left(j2\pi F_{D}s\right)z_{Q}^{*}\left(t-s\right)ds,$$

where the superscript “*” denotes complex conjugation. Replacing the transmitted pulse train $z_{P}\left(t\right)$ by the return from the target $r_{P}\left(t\right)$, gives $\chi_{PQ}\left(t,F_{D}\right)$, which provides the delay and Doppler of targets in a Delay–Doppler map [5,37].

First order Reed–Müller codes RM(1,N) have been used to code the transmission order of Golay complementary waveforms. Suitable assignment of the transmission order using Reed–Müller codes suppresses the range sidelobes of AF at a Doppler shift of interest [34]. These first order Reed–Müller codes can be generated by a Walsh matrix of order $2^{M}$, denoted by $W_{2^{M}}$, where $N=2^{M}\left(M,N\in N\right)$. If the designed parameter of pulse number $N<N_{0}<2^{M+1}$, then we should use the first *N* pulses for the following optimal transmission order selection criterion:

(6) $$W_{2^{m+1}}=\left[\begin{array}{cc} W_{2^{m}} & W_{2^{m}}\\ W_{2^{m}} & -W_{2^{m}} \end{array}\right],\;m=0,1,\ldots ,M-1,$$

where $W_{2^{0}}=1$.

After a pointwise operation $(W_{2^{M}}+1)/2$ to the Walsh matrix, each row (or column, since the Walsh matrix is symmetric) of the output matrix denotes a transmission order of the Golay complementary waveforms, or, in other words, denotes a *P* sequence. The resulting binary Walsh matrix provides a transmission order library and, for a fixed Doppler of interest in the AF, we can choose the optimal row to determine transmission to achieve the lowest range sidelobes near that Doppler bin [34]. Notice that, since both x(l) and y(l) contain the same energy, the transmission energies of the pulse trains ordered by each row of the Walsh matrix are the same. The criterion for optimal transmission order selection is described next.

Let $F_{D}$ represent the target Doppler measured in Hz, and note that it can also be represented in phase [1], that is $\theta_{1}=F_{D}\left(2\pi T\right)$, where the unit is rad. If $\theta_{1}\notin \left[0,2\pi \right]$rad, then $\theta_{1}\;\mathrm{mod}\;\pm$2π until $\theta_{1}\in \left[0,2\pi \right]$rad. Next, we construct a binary sequence $\left[a_{M},a_{M-1},\ldots ,a_{1}\right]$, starting with $a_{1}$:

(7) $$a_{b}=\left\{\begin{array}{cc} 1, & \mathrm{if}\;\theta_{b}\in \left[0,\;\pi /2\right]\;\cup \;\left[3\pi /2,\;2\pi \right]\;\mathrm{rad},\\ 0, & \mathrm{otherwise}. \end{array}\right.$$

From b=2, we replace $\theta_{b}=2\theta_{b-1}$ to calculate $a_{b},\;b=2,\ldots ,M$ according to Equation (7). Note that $\theta_{b}$ should be “modded” into [0,2π] rad. Repeat the above process until $\theta_{M}$ is reached and then the row index *x* is calculated:

(8) $$x=\sum_{b=1}^{M}2^{b-1}a_{b}.$$

**Figure 1:**
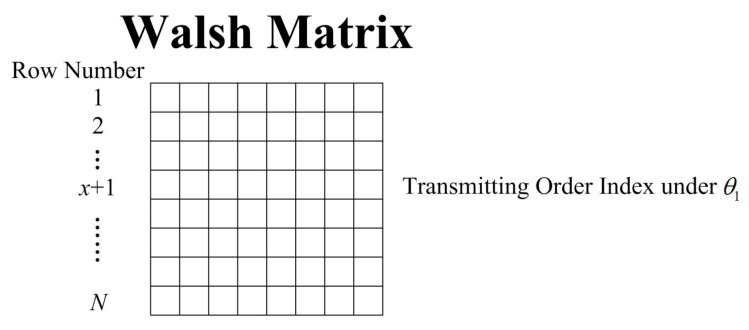
The schematic figure of the selected row in Walsh matrix for a given Doppler value.

The optimal transmission order of the Golay complementary waveforms that minimizes range sidelobes on the $\theta_{1}$-Doppler line is represented by the (x+1)th row of the Walsh matrix. A schematic is given in Figure 1 to illustrate the selected row for $\theta_{1}$.

## 3. The Proposed Signal Processing Procedure

**Figure 2:**
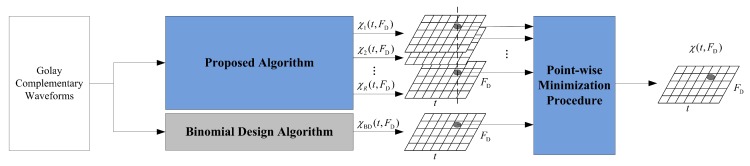
The schematic figure of the proposed signal processing procedure.

We now briefly describe the Binomial Design algorithm proposed in [32,33], in which a standard Golay complementary waveform sequence is transmitted, but weights are assigned to the received pulses. In parallel to this process, another independent Golay complementary waveform sequence is transmitted according to the order determined by one of three proposed transmission order algorithms described in the follow sub-sections. A pointwise nonlinear procedure is then applied to combine the Delay–Doppler maps from the two processes to obtain the final Delay–Doppler map. A schematic of this signal processing procedure is shown in Figure 2, where *R* is the number of generated Delay–Doppler maps for each ordering algorithm. We will then discuss each algorithm in detail. This signal processing procedure is expected to further reduce range sidelobes along given Doppler lines. Note that the Dopplers of the targets are required in Algorithms 2 and 3, and it may be estimated, for example, from the output of a Doppler (for instance, continuous wave) radar, or by a tracker prediction (such as a Kalman filter) from past detections [38]. Sections 2.3.3 and 2.3.4 in [39] provide an example to show a method in detail for the estimation of the time delay (for range sidelobe regions estimation in Section IV) as well as the Doppler shifts of the targets under Golay complementary waveforms.

### 3.1. Binomial Design Algorithm

**Figure 3:**
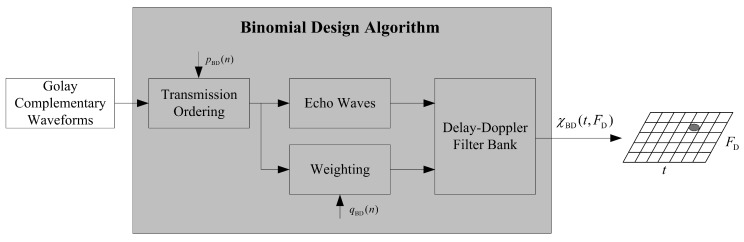
Signal processing structure of the Binomial Design algorithm.

**Figure 4:**
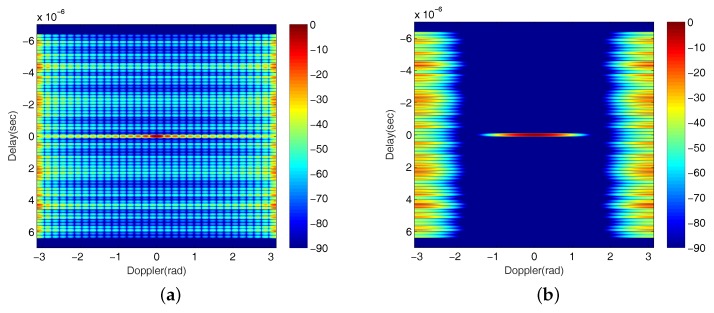
The AF of Golay complementary waveforms based on (**a**) standard transmission order; (**b**) Binomial Design algorithm (the unit of colorbar is dB).

In this algorithm, the *P* sequence represents the standard transmission order of Golay complementary waveforms and the *Q* sequence satisfies ${\left\{q(n)\right\}}_{n=0}^{N-1}=\alpha {\left\{C_{N-1}^{n}\right\}}_{n=0}^{N-1}$, where $\alpha =N/\sum_{n=0}^{N-1}C_{N-1}^{n}$ is a normalizing factor. Figure 3 shows the flowchart of the Binomial Design algorithm, where the returns of the transmitted Golay complementary waveforms are processed by a matched filter where the filtering is a done by a copy of the transmitted pulse sequence weighted by the *Q* sequence. Then, $\chi_{\mathrm{BD}}\left(t,F_{D}\right)$ represents the Delay–Doppler map obtained through the Binomial Design algorithm. As to this algorithm, lower order terms in the Taylor Series of the $\chi_{\mathrm{BD}}\left(t,F_{D}\right)$ (expanded according to the Doppler $F_{D}$) at $F_{D}=0$ are killed, and this has the effect of reducing sidelobes close to the zero-Doppler line. Increasing the number of terms in the Taylor Series that are nulled by this process will also increases the width of the sidelobe blanking area in the AF. The use of *N* pulses for this algorithm provides N−2 zeros in the Taylor Series, which yields a sidelobe blanking area (that is at less than −90dB), approximately [−π,π] (radians), in the AF when N=16 [32]. Note that larger *N* will result in a better match filtering performance, which increases the magnitude difference between the mainlobe and sidelobes shown in the AF and thus expands the sidelobe blanking area. However, as mentioned in Section 1, this algorithm gives inferior Doppler resolution. In addition, it is clear that the Binomial Design algorithm transmits and receives the same waveform energy as the standard Golay complementary waveforms. As indicated in [1], from Moyal’s identity, the AF has an energy only dependent on the energy of the waveform, which means the algorithm actually achieves a significant range sidelobe blanking area by pushing range sidelobes energy into other (hopefully, uninteresting) areas in the Delay–Doppler map. Clearly, raising the magnitude of range sidelobes in the area where target is presented may lead to target miss detection, so that, if another weak target falls in the sidelobes of a strong target, a missed detection of the weak target may occur. Figure 4 provides a deeper understanding to the difference between the Golay complementary waveforms based on standard transmission order and Binomial Design algorithm (the parameters setting of this figure are described in Section 5). The drawbacks of the Binomial Design algorithm can be addressed by the signal processing procedure proposed in this work.

### 3.2. Algorithm 1

Previous discussion describes how the approach of Suvorova et al. [34] actually uses a finite library of transmission orders to cover all (quantized) Doppler values in the Delay–Doppler map. The Golay complementary waveform ordering by each row of the Walsh matrix minimizes the range sidelobes with respect to a particular Doppler bin. Increasing the number of pulses *N* lead to smaller Doppler bins and reduced magnitude of range sidelobes. The best performance can be expected, then, if we transmit the Golay complementary waveforms using every row of the Walsh matrix in parallel and combine the processing results somehow; this is termed *Algorithm 1*. As we will discuss later, one way to combine multiple processing results is to use a pointwise minimization. Nevertheless, Algorithm 1 needs to transmit *N* series of Golay complementary waveforms pulse trains, and each pulse train contains *N* pulses with a different transmission order. It will output *N* Delay–Doppler maps to the ensuing nonlinear processing. Let *R* in Figure 2 be equal to *N*, then Figure 2 represents the processing structure of Algorithm 1, and we denote $\chi 1_{1}\left(t,F_{D}\right)∼\chi 1_{N}\left(t,F_{D}\right)$ as the Delay–Doppler maps obtained through Algorithm 1.

### 3.3. Algorithm 2

While Algorithm 1 suppresses the range sidelobes for a Delay–Doppler map by transmitting Golay complementary waveforms in all orders given by rows of the Walsh matrix, it also yields the longest transmission time and maximum computational complexity in the three ordering algorithms (detailed discussion can be seen in Section 4). This complexity can be reduced by transmitting only the rows which correspond to those Doppler shifts of the underlying targets, and this is called *Algorithm 2*. Figure 2 represents the processing structure of Algorithm 2 when R=H, where *H* is the number of targets with different Doppler, and we write $\chi 2_{1}\left(t,F_{D}\right)∼\chi 2_{H}\left(t,F_{D}\right)$ for the Delay–Doppler maps obtained through Algorithm 2. If the number of targets with different Doppler values is not less than the total number of rows of the Walsh matrix, i.e., H⩾N, Algorithm 2 is identical to Algorithm 1.

### 3.4. Algorithm 3

*Algorithm 3* uses a weighted mean Doppler from the Doppler shifts of all targets presented in the Delay–Doppler map to select the optimal row of the Walsh matrix for the transmission order of Golay complementary waveforms. Obviously, this algorithm attempts to strike a balance between computational complexity of the algorithm and range sidelobe suppression performance. The weighted mean Doppler for all targets presented is calculated as

(9) $${\bar{f}}_{d}=\left\{\begin{array}{cc} \frac{\sum_{h=1}^{H}f_{d_{h}}}{H}, & \;\mathrm{if}\;\mathrm{all}\;A_{h}\;\mathrm{are}\;\mathrm{the}\;\mathrm{same},\\ \frac{\sum_{h=1}^{H}\left(1-A_{h}\right)f_{d_{h}}}{\sum_{h=1}^{H}\left(1-A_{h}\right)}, & \;\mathrm{otherwise}. \end{array}\right.$$

where $A_{h}$ and $f_{d_{h}}$ are the normalized magnitude and the Doppler of the hth target, respectively, and h=1,2,…,H. The optimal transmission order in the library that achieves the best sidelobe suppression performance is selected according to ${\bar{f}}_{d}$. Let *R* in Figure 2 be equal to 1, then Figure 2 represents processing structure of Algorithm 3, and we write $\chi 3_{{\bar{f}}_{d}}\left(t,F_{D}\right)$ for the Delay–Doppler map obtained using Algorithm 3. Since it involves a trade-off of sidelobe suppression performance and computational complexity, Algorithm 3 is more computationally efficient than Algorithms 1 and 2 but with a higher false-alarm rate compared with the other two algorithms.

### 3.5. Pointwise Minimization

As mentioned earlier, the Binomial Design algorithm achieves significantly lower range sidelobes for targets near zero-Doppler by considerably sacrificing Doppler resolution. On the other hand, the three ordering algorithms achieve better Doppler resolution performance compared to the Binomial Design algorithm, though they yield a smaller sidelobe blanking area. We propose, then, to use a pointwise minimization procedure to combine the outputs from the two independent processes, taking advantage of both the Binomial Design algorithm and one of the ordering algorithms. The operation is mathematically expressed as follows:

(10) $$\begin{array}{cc} \chi 1(t,F_{D})= & \min[\chi 1_{1}\left(t,F_{D}\right),\ldots ,\chi 1_{u}\left(t,F_{D}\right),\ldots ,\\ & \chi 1_{N}\left(t,F_{D}\right),\chi_{\mathrm{BD}}\left(t,F_{D}\right)], \end{array}$$

(11) $$\begin{array}{cc} \chi 2(t,F_{D})= & \min[\chi 2_{1}\left(t,F_{D}\right),\ldots ,\chi 2_{h}\left(t,F_{D}\right),\ldots ,\\ & \chi 2_{H}\left(t,F_{D}\right),\chi_{\mathrm{BD}}\left(t,F_{D}\right)], \end{array}$$

(12) $$\chi 3\left(t,F_{D}\right)=\min\left[\chi 3_{{\bar{f}}_{d}}\left(t,F_{D}\right),\chi_{\mathrm{BD}}\left(t,F_{D}\right)\right],$$

where $\chi 1(t,F_{D})$, $\chi 2(t,F_{D})$ and $\chi 3(t,F_{D})$ are the final output Delay–Doppler map of Algorithms 1–3, respectively. The proposed pointwise minimization can also be studied in Figure 2. Based on the assumption that targets are presented at a constant delay and Doppler during radar illumination, the location and magnitude of corresponding targets will be the same in all the Delay–Doppler maps of each algorithm, while sidelobes are different in each map. After the pointwise minimization, the point in the final Delay–Doppler map that represents the target retains a high magnitude while all other points are significantly reduced. This results in the expansion of range sidelobe blanking area and the preservation of Doppler resolution. In practical experiments, however, or even simulations, the location of targets may be offset in each Delay–Doppler map because of micro-motion or the overlap influence of range sidelobes and noise, which leads to the fluctuating of effective target magnitude. It is certain that if the offset of target location is too large, the pointwise minimization may also blank the target. In the next section, we will further discuss the effectiveness of the pointwise minimization, and provide a quantitative analysis on the tolerable location offset region of the target in the Delay–Doppler map that can continue to achieve an acceptable performance. In addition, the statistical simulation results under the Swerling II target detection presented in Section 5 is also deemed an effective justification of this nonlinear operation.

## 4. Analysis of the Proposed Signal Processing Procedure

Here, we analyze the performance of the proposed signal processing procedure for radar detection using Golay complementary waveforms in the four algorithms above. Firstly, the computational complexity of the Binomial Design algorithm and the proposed three ordering algorithms described in the last section are examined; we then study the condition at which the pointwise minimization can yield acceptable performance; thirdly, a method to estimate the areas in the Delay–Doppler map taken by range sidelobes based on knowledge of presented target is described.

### 4.1. Computational Complexity Comparison

Derived from the definition in Equation (5), the AF under the Binomial Design algorithm is given by Equation (13),

(13) $$\begin{array}{cc} \chi_{\mathrm{BD}}\left(t,F_{D}\right) & =\int_{-\infty }^{+\infty }z_{P_{\mathrm{BD}}}\left(s\right)\exp\left(j2\pi F_{D}s\right)z_{Q_{\mathrm{BD}}}^{*}\left(t-s\right)ds\\ = & \sum_{k=-L+1}^{L-1}\sum_{n=0}^{N-1}q_{\mathrm{BD}}\left(n\right)\exp\left(j2\pi F_{D}nT\right)\\ & \times [p_{\mathrm{BD}}\left(n\right)x\left(t-nT\right)x^{*}\left(t-nT\right)\\ & +\left(1-p_{\mathrm{BD}}\left(n\right)\right)y\left(t-nT\right)y^{*}\left(t-nT\right)]\\ = & \sum_{k=-L+1}^{L-1}\sum_{n=0}^{N-1}q_{\mathrm{BD}}\left(n\right)\exp\left(j2\pi F_{D}nT\right)\\ & \times [p_{\mathrm{BD}}\left(n\right)C_{x}\left(k\right)+\left(1-p_{\mathrm{BD}}\left(n\right)\right)C_{y}\left(k\right)]\\ & \times \Omega \left(t-kT_{c}-nT\right)\Omega^{*}\left(t-kT_{c}-nT\right)\\ = & \frac{1}{2}\sum_{k=-L+1}^{L-1}\left[C_{x}\left(k\right)+C_{y}\left(k\right)\right]\sum_{n=0}^{N-1}q_{\mathrm{BD}}\left(n\right)\\ & \times \exp\left(j2\pi F_{D}nT\right)C_{\Omega }\left(t-kT_{c}-nT\right)\\ & -\frac{1}{2}\sum_{k=-L+1}^{L-1}\left[C_{x}\left(k\right)-C_{y}\left(k\right)\right]\sum_{n=0}^{N-1}{\left(-1\right)}^{p_{\mathrm{BD}}\left(n\right)}\\ & \times q_{\mathrm{BD}}\left(n\right)\exp\left(j2\pi F_{D}nT\right)C_{\Omega }\left(t-kT_{c}-nT\right), \end{array}$$

where $p_{\mathrm{BD}}\left(n\right)$ and $q_{\mathrm{BD}}\left(n\right)$ represent the (n+1)th values of the *P* and *Q* sequences for the Binomial Design algorithm, $C_{\Omega }\left(t\right)$ is the autocorrelation of Ω(t). As expressed in Equation (1), the first term in the final line of calculation of $\chi_{\mathrm{BD}}\left(t,F_{D}\right)$ does not contain any range sidelobes and controls the peak in the AF; in other words, it is the main contributing factor to the target information in the Delay–Doppler map. The second term generates the range sidelobes in the AF, with energy controlled by the values of $p_{\mathrm{BD}}\left(n\right)$ and $q_{\mathrm{BD}}\left(n\right)$.

The computational complexity of an algorithm is the number of elementary arithmetic operations in the algorithm computation. The computational complexity of $\chi_{\mathrm{BD}}\left(t,F_{D}\right)$ can be divided into three parts. The first is the calculation of $C_{x}\left(k\right)$ and $C_{y}\left(k\right)$, resulting in a computational complexity of 2L−1 for each. The second part is the calculation of $\exp\left(j2\pi F_{D}nT\right)C_{\Omega }\left(t-kT_{c}-nT\right)$. With *X* and *Y* sampling points along time the delay and Doppler axes respectively, the computational complexity is (2X−1)Y. The third part is the summation over *N* pulses, which yields a multiplier to the computational complexity of *N* times. The total computational complexity for the Binomial Design algorithm is, then, (4L−2)+(2X−1)YN.

Similarly, for Algorithm 1, we have

(14) $$\begin{array}{cc} \chi 1_{u}\left(t,F_{D}\right) & =\frac{1}{2}\sum_{k=-L+1}^{L-1}\left[C_{x}\left(k\right)+C_{y}\left(k\right)\right]\sum_{n=0}^{N-1}q_{1_{u}}\left(n\right)\\ & \times \exp\;\left(j2\pi F_{D}nT\right)C_{\Omega }\left(t-kT_{c}-nT\right)\\ & -\frac{1}{2}\sum_{k=-L+1}^{L-1}\left[C_{x}\left(k\right)-C_{y}\left(k\right)\right]\sum_{n=0}^{N-1}{\left(-1\right)}^{p_{1_{u}}\left(n\right)}\\ & \times q_{1_{u}}\left(n\right)\exp\left(j2\pi F_{D}nT\right)C_{\Omega }\left(t-kT_{c}-nT\right), \end{array}$$

where ${\left\{p_{1_{u}}\left(n\right)\right\}}_{n=0}^{N-1}$ and ${\left\{q_{1_{u}}\left(n\right)\right\}}_{n=0}^{N-1}$ represent the *P* and *Q* sequences of $\chi 1_{u}\left(t,F_{D}\right)$, the subscript “*u*” signifying the uth Delay–Doppler map. In a similar manner, the expressions for Algorithms 2 and 3 are

(15) $$\begin{array}{cc} \chi 2_{h}\left(t,F_{D}\right) & =\frac{1}{2}\sum_{k=-L+1}^{L-1}\left[C_{x}\left(k\right)+C_{y}\left(k\right)\right]\sum_{n=0}^{N-1}q_{2_{h}}\left(n\right)\\ & \times \exp\left(j2\pi F_{D}nT\right)C_{\Omega }\left(t-kT_{c}-nT\right)\\ & -\frac{1}{2}\sum_{k=-L+1}^{L-1}\left[C_{x}\left(k\right)-C_{y}\left(k\right)\right]\sum_{n=0}^{N-1}{\left(-1\right)}^{p_{2_{h}}\left(n\right)}\\ & \times q_{2_{h}}\left(n\right)\exp\left(j2\pi F_{D}nT\right)C_{\Omega }\left(t-kT_{c}-nT\right) \end{array}$$

and

(16) $$\begin{array}{cc} \chi 3_{{\bar{f}}_{d}}\left(t,F_{D}\right) & =\frac{1}{2}\sum_{k=-L+1}^{L-1}\left[C_{x}\left(k\right)+C_{y}\left(k\right)\right]\sum_{n=0}^{N-1}q_{3_{{\bar{f}}_{d}}}\left(n\right)\\ & \times \exp\left(j2\pi F_{D}nT\right)C_{\Omega }\left(t-kT_{c}-nT\right)\\ & -\frac{1}{2}\sum_{k=-L+1}^{L-1}\left[C_{x}\left(k\right)-C_{y}\left(k\right)\right]\sum_{n=0}^{N-1}{\left(-1\right)}^{p_{3_{{\bar{f}}_{d}}}\left(n\right)}\\ & \times q_{3_{{\bar{f}}_{d}}}\left(n\right)\exp\left(j2\pi F_{D}nT\right)C_{\Omega }\left(t-kT_{c}-nT\right). \end{array}$$

Note that the subscript “*h*” in Equation (15) indicates the hth Delay–Doppler map in Algorithm 2, and “${\bar{f}}_{d}$” in Equation (16) denotes the Delay–Doppler map in Algorithm 3 based on the weighted mean Doppler ${\bar{f}}_{d}$. Clearly, $\chi 1_{u}\left(t,F_{D}\right)$, $\chi 2_{h}\left(t,F_{D}\right)$ and $\chi 3_{{\bar{f}}_{d}}\left(t,F_{D}\right)$ are of the same computational complexity as $\chi_{\mathrm{BD}}\left(t,F_{D}\right)$.

Based on the signal processing procedures in Figure 2, the comparison of computational complexity of the algorithms is presented in Table 1. For each of the three algorithms, XY is the computational complexity of the pointwise minimization. Table 1 indicates that the total computational complexity of Algorithms 1–3 are about (N+1)×, (H+1)× and 2× compared to that of the Binomial Design algorithm, respectively.

### 4.2. Performance Analysis of the Pointwise Minimization

The pointwise minimization in the proposed signal processing procedure is an information loss process. After the pointwise minimization, whether signals carrying target information remain present in the Delay–Doppler map is of concern. As mentioned earlier, we assume that the underlying targets remain in the scene at constant delay and Doppler during the entire radar illumination period. This means that, if a target return is present in one of the Delay–Doppler maps, it will also appear in all other Delay–Doppler maps. The following causes of uncertainty can cause errors in the precise location and signal magnitude of the target:

1. target return fluctuations because of target micro-motion (as modelled by the Swerling II target model);
2. the presence of range sidelobes, many of which have strong magnitude and may give rise to false targets.

We wish to understand the conditions under which target detection from the Delay–Doppler map can be guaranteed in the presence of these uncertainties. We focus, first, on the first problem, i.e., target return fluctuations. The second problem is covered in the next sub-section. We define a statistically minimal region, called the “tolerable location offset region (TLOR)” denoted by *O*, so that target returns under the Swerling II model will overlap across all Delay–Doppler maps sufficiently for the pointwise minimization process to retain detection. A threshold can be found to achieve a very high detection probability of a target if the target return falls into the TLOR.

**Figure 5:**
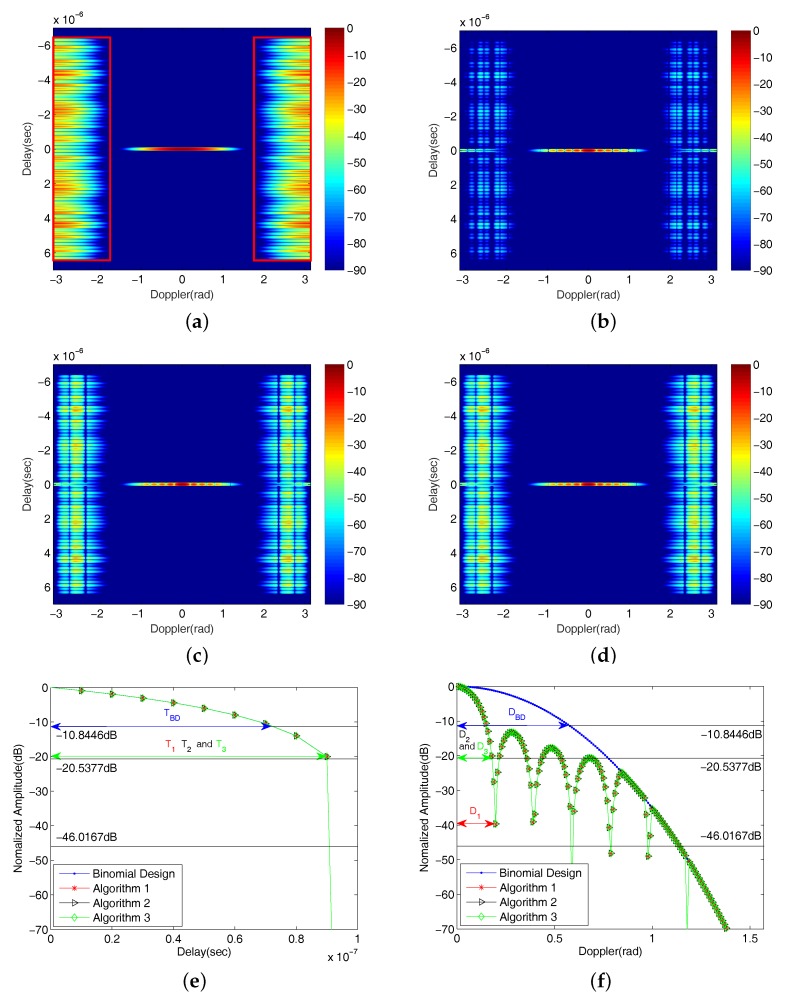
The AFs of (**a**) Binomial Design algorithm; (**b**) Algorithm 1; (**c**) Algorithm 2; (**d**) Algorithm 3; and the comparisons of (**e**) delay mainlobes/zero-Doppler cross section; and (**f**) Doppler mainlobes/zero-delay cross section (the unit of colorbar is dB).

As an example, we plot the AFs of the Binomial Design algorithm and the three ordering algorithms and compare their delay and Doppler mainlobes in Figure 5, where parameters used for the plots are given in Section 5. Recall that, in practice, this procedure works in the context of a tracker, where prior information is available about the location of targets.

From Figure 5, the following observations can be made:

1. The three ordering algorithms have much lower range sidelobes and higher Doppler resolutions than Binomial Design algorithm.
2. The results of Figure 5c,d are the same in the single target situation. Note that Algorithm 2 will perform better than Algorithm 3 in multiple Doppler targets case, at the cost of a higher computational complexity.

To ensure that the target signal remains in the Delay–Doppler map after the pointwise minimization, we consider the worst case that the magnitude of the target signal may be reduced, but the magnitude of range sidelobes remains at the same level as before the pointwise minimization. We should find a TLOR *O* centered at the ground truth location of target signal such that the following inequality holds:

(17) $$\frac{\chi \left(t,F_{D}\right){|}_{(t,F_{D})\in O}}{\max_{\left(t,F_{D}\right)\in S_{d}}\;\chi \left(t,F_{D}\right)}>1,$$

where $S_{d}$ signifies the areas that contain significant range sidelobes (assumed known in our simulation), thresholded at a magnitude DL (throughout the simulations in our work, we set DL=−90dB), $(t,F_{D})\in O$ represents an arbitrary point in TLOR, or, in other words, one of the possible locations of the target signal after the pointwise minimization, and $\chi \left(t,F_{D}\right){|}_{(t,F_{D})\in O}$ signifies the signal magnitude at that point. Inequality (17) claims that target in *O* will still be detected since its magnitude is larger than the maximum magnitude of the range sidelobes after the pointwise minimization. In practice, it may lose the target if its signal is only a little larger than the maximum magnitude of the range sidelobes because of the influence of noise, and so we limit the target signal to be at least 3 dB larger than the maximum range sidelobe level in the simulation to guarantee the detection of the target.

**Figure 6:**
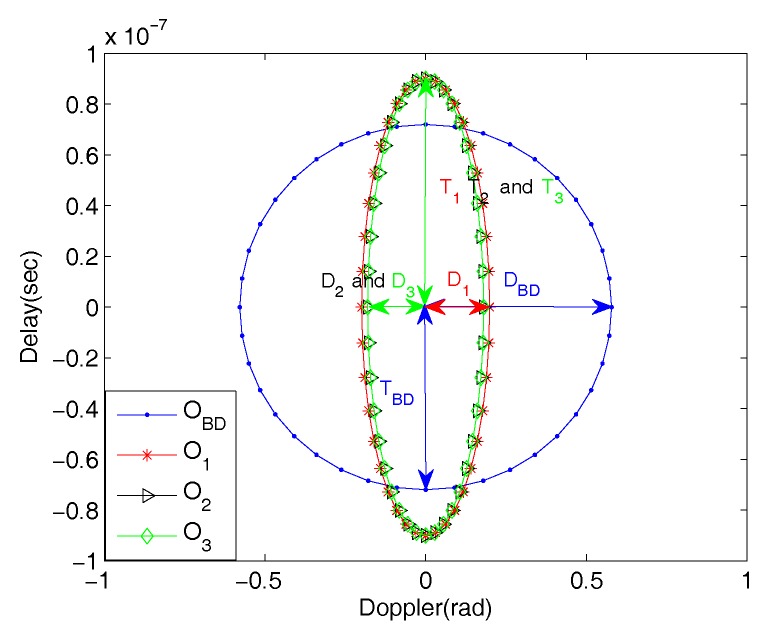
Illustration of the TLOR $O_{i}$, i=BD,1,2,3. under corresponding algorithms. Each $\left(T_{i},D_{i}\right)$ defines the TLOR under the Algorithm *i*.

We use the results in Figure 5 to illustrate the TLOR *O*. Firstly, the largest range sidelobe magnitudes for the Binomial Design algorithm and the three ordering algorithms are found to be −13.8446dB, −49.0167dB, −23.5377dB and −23.5377dB, respectively. Secondly, each of these values (plus 3dB) is used as a cut off threshold for TLOR in the delay and Doppler mainlobes under each of the algorithms as indicated in Figure 5e,f. The TLOR generates an ellipse centered at the ground truth location of the target signal with different values of delay and Doppler semi-axes listed in Table 2. In Figure 6, we further illustrate TLOR $O_{i}$, i=BD,1,2,3 for each of algorithms. Our simulation in Section 5 indicates that, under the Swerling II model, the underlying target signal after pointwise minimization will fall in the corresponding TLOR.

In summary, we conclude this analysis by making the following remarks:

1. *O* is defined by Equation (17) as a TLOR in the Delay–Doppler map centered at the ground truth location of the target signal, with size determined by the boundary of delay and Doppler mainlobes and the maximum magnitude of range sidelobes. The target signal in TLOR remains present after the pointwise minimization.
2. In the presence of multiple nonzero Doppler targets, $S_{d}$ and *O* can be partially overlapped. However, as long as the target magnitude at *O* is at least 3 dB larger than the maximum magnitude of sidelobes, the pointwise minimization will not lose the target.
3. If the maximum magnitude of sidelobes is less than the targets’ mainlobes, the TLOR can be approximated by the boundary of the mainlobes.
4. In practice, the previously estimated target location obtained from a tracker is used to replace the ground truth location as the center of *O*.
5. We note that the determination of the TLOR in the above simulations is based on knowledge of target location, magnitude and sidelobe level. These are hard to find in a realistic situation. These simulations are used to illustrate the tolerance of the pointwise minimization to fluctuations of in target returns, and to do performance analysis. Determination of actual sidelobe and sidelobe regions in practice still needs further study.

**Figure 7:**
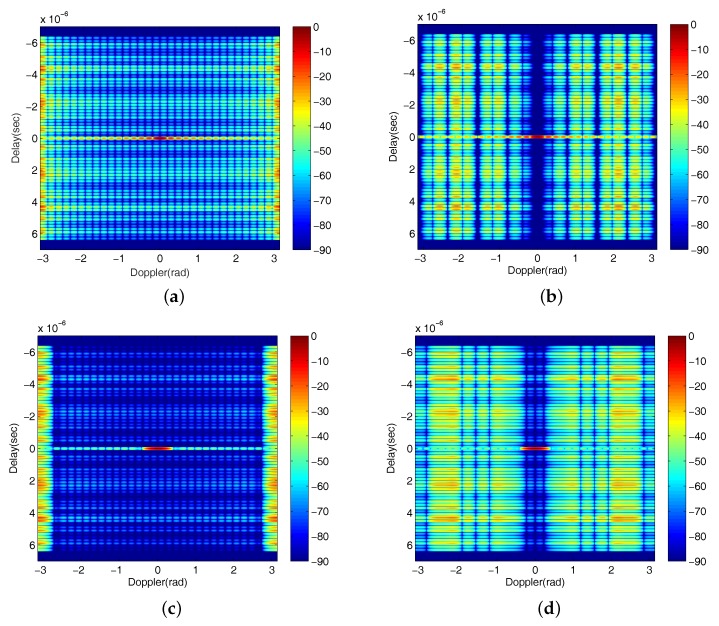
The AFs of some representative existing waveform design methods for Golay complementary waveforms (the unit of colorbar is dB). (**a**) Standard transmission order; (**b**) PTM Design algorithm; (**c**) Hamming amplitude weighting; (**d**) both PTM Design algorithm and Hamming amplitude weighting applied.

As aforementioned, there are also a number of existing methods under Golay complementary waveforms for range sidelobe suppression. In Figure 7, we plot the results generated by some representative methods for a comparison. Figure 7a is the result of standard transmission order with no receiving weights (which is identical to the Figure 4a); Figure 7b demonstrates the result of using the Prouhet–Thue–Morse (PTM) Design algorithm shown in [23]; Figure 7c shows the AF of using a Hamming amplitude weighting [36] to the Golay complementary waveforms, and it has a similar performance as the Binomial Design algorithm (shown in Figure 4b) in that it sacrifices the Doppler resolution for a larger sidelobe blanking area. Figure 7d shows the result for applying both PTM Design algorithm and Hamming amplitude weighting. Clearly, the performance is poorer than that by just using one of them. It is worth mentioning that the approaches proposed in this work are different from all the methods in Figure 7, and their AFs given in Figure 5b–d suggest an improved performance over these comparison methods.

### 4.3. Estimation of Significant Range Sidelobe Regions in Delay–Doppler Map

Estimation of the significant range sidelobe regions in the Delay–Doppler map for given targets arising from the processing of Golay complementary waveforms is an important factor in the separation of targets from sidelobes. In our following simulations, we still set the sidelobe threshold at DL=−90dB. Since the three ordering algorithms can be implemented in parallel with the Binomial Design algorithm, followed by a pointwise minimization (See Figure 2), the range sidelobe areas in the output Delay–Doppler maps will be the same as the Binomial Design algorithm for all the three of the ordering algorithms (an example can be seen in Figure 5), so we only need to consider the Binomial Design algorithm to estimate the range sidelobes in the output Delay–Doppler map.

**Figure 8:**
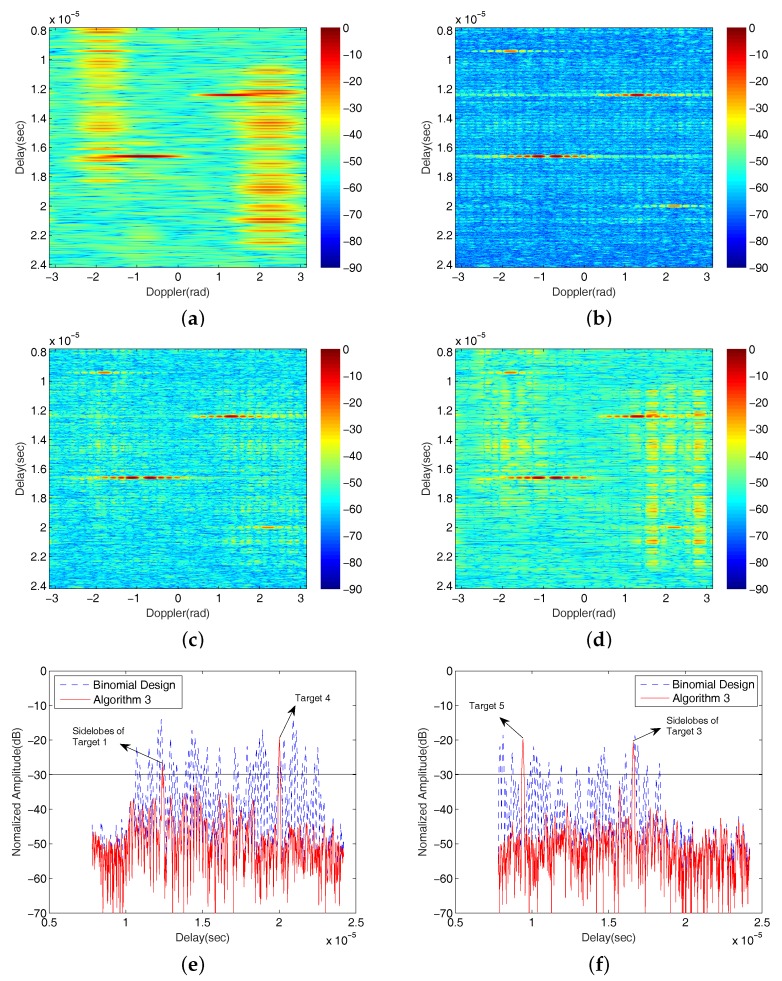
The output Delay–Doppler maps of (**a**) Binomial Design algorithm; (**b**) Algorithm 1; (**c**) Algorithm 2; (**d**) Algorithm 3 in the particular set target detection scene; and the comparisons of Doppler cross sections of the weak targets (**e**) Target 4; (**f**) Target 5 for Binomial Design algorithm and Algorithm 3 (the unit of colorbar is dB).

As shown in Figure 5a, the length of sidelobe areas along the Delay axis is $\left[-LT_{c},LT_{c}\right]$, a consequence of matched filtering in the time domain. However, finding the length of sidelobe areas along the Doppler axis is non-trivial. For a given pulse number *N*, the size of the thresholded range sidelobes is clearly a function of the threshold DL. In Figure 5 and the following Figure 8, only range sidelobes larger than DL=−90dB are displayed at their original magnitudes. All range sidelobes less than this are displayed as −90dB. A larger DL clearly yields a wider range sidelobe blanking area, which shrinks the thresholded regions in the Delay–Doppler map. Nevertheless, the following method can be adopted to estimate the length of sidelobe areas along the Doppler axis. Assuming that the length of sidelobe areas along the Doppler axis is $\left[-2\pi ,-f_{0}\right]\cup \left[f_{0},2\pi \right]$rad in the AF, then the value of $f_{0}$ satisfies the following condition:

(18) $$f_{0}=\underset{F_{D}\in \left[-2\pi ,2\pi \right]}{\arg\max}\left\{\chi_{\mathrm{BD}}\left(:,\;F_{D}\right)\right\}\;<DL.$$

Inequality (18) indicates that the magnitudes along the Doppler boundary $f_{0}$ are less than DL. For example, the sidelobe areas in Figure 5a when DL=−90dB is expressed as

[−6.4,6.4]μs×[−2π,−1.783]∪[1.783,2π]rad.

These areas are illustrated using red rectangles in Figure 5a. In the presence of multiple nonzero Doppler targets, sidelobe areas are also influenced by target locations in the Delay–Doppler map (again, a tracker is needed to estimate target locations). Assuming the estimated location of the hth target in the Delay–Doppler map is $\left(\tau_{h},f_{d_{h}}\right)$, then the sidelobe areas are given by

(19) $$\overset{H}{\underset{h=1}{⋃}}\left\{\left[\tau_{h}-LT_{c},\tau_{h}+LT_{c}\right]\times \left[-2\pi ,f_{d_{h}}-f_{0}\right]\cup \left[f_{d_{h}}+f_{0},2\pi \right]\right\}.$$

In summary, regarding the estimation of the thresholded (at DL) range sidelobe regions for a specified algorithm, it is required to know estimated target locations (as given by a tracker) $\left(\tau_{h},f_{d_{h}}\right)$, number of pulses *L* and chip interval $T_{c}$ in the Golay complementary waveforms as well as the threshold DL.

## 5. Numerical Simulations and Discussion

In this section, we present numerical simulations to demonstrate the effectiveness of the proposed three ordering algorithms and verify the analytical results. The global simulation parameters are as follows. We assume that the radar carrier frequency is $f_{c}=1\;\mathrm{GHz}$, bandwidth is B=50MHz, sampling rate is $f_{s}=2B$, PRI is T=50μs, number of pulses $N=2^{5}=32$. For the Golay complementary waveforms, the number of chips is L=64 with values ±1 and the chip interval is $T_{c}=0.1\;\;\;\;\mu \;\;\;s$. Each chip has $f_{s}\times T_{c}=10$ sampling points.

### 5.1. Simulation under a Fixed Scenario

**Figure 9:**
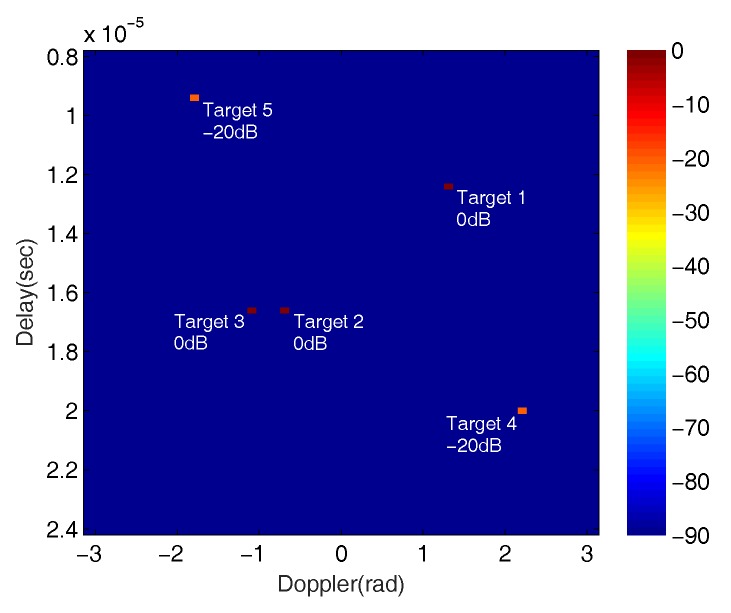
Illustration of target signal magnitudes and locations in the Delay–Doppler map for the fixed scenario.

The fixed scenario contains five targets, three strong targets (Targets 1–3) with normalized signal magnitudes 0dB and 2 weak targets (Target 4∼5) at −20dB, as shown in Figure 9.

The locations of these targets in the Delay–Doppler map are listed in Table 3.

While other targets can be resolved in both delay and Doppler differences, Targets 2 and 3 can only be distinguished by their Doppler values. These targets are simulated using a Swerling II target model with parameter $\sigma^{2}=0.3$ [40]. We also set a maximum 10% fluctuation of each delay and Doppler, relative to the original values from pulse to pulse. The radar returns at the receiver are contaminated by a complex Gaussian zero-mean white noise E∼CN(0,1) with mean magnitude of −10dB (i.e., SNR=10dB).

The simulation results of the Binomial Design algorithm, and Algorithms 1–3 are shown in Figure 8. It is observed in Figure 8a that the Binomial Design algorithm has a poor target detection performance in the presence of multiple nonzero Doppler targets. The two weak targets are both masked by the sidelobes of strong targets and are undetectable. In addition, Targets 2 and 3 are not separated because of the low Doppler resolution of this algorithm. On the other hand, in the Delay–Doppler maps of the three ordering algorithms shown in Figure 8b–d, Targets 2 and 3 can be visually separated.

Figure 8e,f show the Doppler cross sections of the two weak targets, Targets 4 and 5, using the Binomial Design algorithm and using Algorithm 3 (since these two targets will clearly be seen using Algorithms 1 and 2, it is not necessary to put these Doppler cross sections in the figures). The weak targets are almost undetectable using the Binomial Design algorithm. On the other hand, a fairly arbitrary threshold of −30dB based on our simulations is set, which enables easier detection of weak targets using Algorithm 3 because of high SNR.

### 5.2. Simulation under a Randomized Scenario

This simulation is intended to statistically compare the detection performances of the algorithms and demonstrate the effectiveness of the proposed signal processing procedures. Without loss of generality, the targets are set to be uniformly distributed in the map and based on the Swerling II model.

The simulation is designed in the following four cases:

1. Two targets are presented in random locations, with one strong target and one weak target;
2. Three targets are presented in random locations, with one strong target and two weak targets;
3. Four targets are presented in random locations, with two strong targets and two weak targets;
4. Five targets are presented in random locations, with three strong targets and two weak targets.

The signal magnitudes of strong target and weak target remain at 0dB and −20dB, respectively. To analyze the detection performance, without loss generality, we use the signal magnitude of the weakest target as the threshold (since during the simulations we exactly know the amplitude and location of all targets) to do the following statistical target detection simulations for each algorithm; this guarantees that in our simulations all the true targets are detected, but false targets are also detected because of the presence of range sidelobes that exceed the threshold. In practice, correct thresholding needs further study. One thousand Monte Carlo runs for each of cases mentioned above are carried out. Note that in these particular simulations we detect every target by comparison with values in a small Doppler interval around its ground truth Doppler, with the length of this Doppler interval set according to the variance of target Doppler estimation through the online tracking system.

The following four measures are used to compare the performances of the four algorithms.

**Figure 10:**
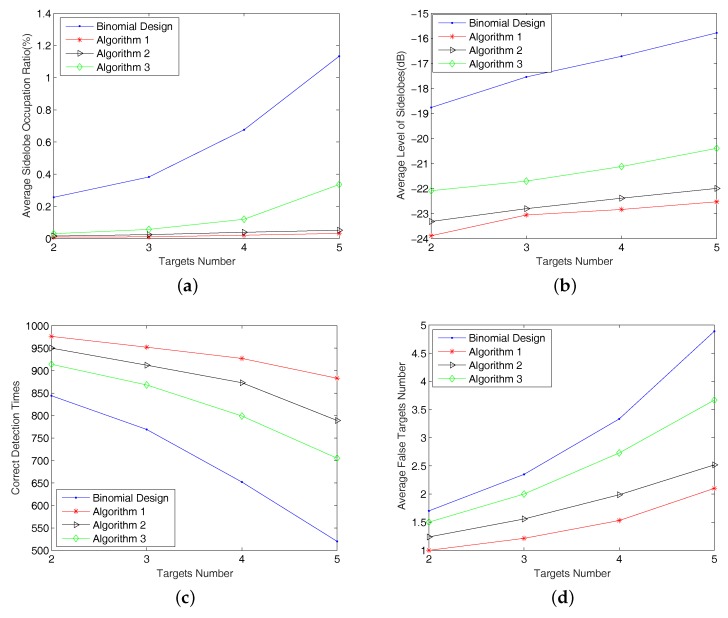
Statistical simulation results of (**a**) average false-alarm sidelobes occupation ratio in the Delay–Doppler map; (**b**) average energy level of false-alarm sidelobes; (**c**) correct detection times; (**d**) average false targets number for the four algorithms.

1. **Average false-alarm sidelobe occupation ratio** in the Delay–Doppler map. We calculate the average ratio of areas of range sidelobes that exceed the threshold and cause false-alarm detection with respect to the entire Delay–Doppler map over all 1000 runs. The simulation result is shown in Figure 10a.
2. **Average magnitude level of false-alarm sidelobes**. We compute the average magnitude of the sidelobes that have caused false detections over all 1000 runs. Simulation results are shown in Figure 10b.
3. **Number of correct detection times**. A correct detection means that all the targets are successfully detected and that there is no false target in a single run. The results of simulations are shown in Figure 10c.
4. **Average number of false targets**: This is the number of false targets appearing in the Delay–Doppler map averaged over all the runs where false target occurs. Results are shown in Figure 10d.

**Discussion:**

- The results shown in Figure 10 indicate that proposed three ordering algorithms have an improved performance compared to the Binomial Design algorithm in terms of the performance measures described above. Algorithm 1 has the best performance among the four algorithms, followed by Algorithm 2, Algorithm 3 and the Binomial Design algorithm.
- The results also verify the effectiveness of the pointwise minimization from a statistical viewpoint.
- As the number of targets presented in the Delay–Doppler map increases, the performance of all algorithms deteriorates and the performance differences between algorithms becomes increasingly significant. Evidently, an increase of range sidelobes in the Delay–Doppler map as the consequence of the presence of more targets causes more false targets.
- The calculation complexity comparison of Binomial Design algorithm, and Algorithms 1–3 in this particular statistical simulation case is 1:33:6:2.

## 6. Conclusions

This paper describes a signal processing procedure for detection of multiple nonzero Doppler targets with a radar using Golay complementary waveforms in Reed–Müller sequences. In addition to use of the existing Binomial Design algorithm for altering the transmission waveform order, we also employ one of the three transmission order design algorithms running in parallel. A pointwise minimization is then used to combine the outputs. Algorithmic computational complexities are analyzed and the performance of pointwise minimization is justified in the sense of tolerable location offset region. The delineation of the areas in the Delay–Doppler map occupied by significant range sidelobes for each of the three ordering algorithms are also discussed. Numerical simulation results in both fixed and randomized target scenarios are presented to demonstrate the effectiveness of the proposed signal processing procedure.

## Figures and Tables

**Table 1:** Comparison of the computational complexity.

Algorithm	Total Computational Complexity
Binomial Design Algorithm	(4L−2)+(2X−1)YN
Algorithm 1	(N+1)[(4L−2)+(2X−1)YN]+XY
Algorithm 2	(H+1)[(4L−2)+(2X−1)YN]+XY
Algorithm 3	2[(4L−2)+(2X−1)YN]+XY

**Table 2:** Comparisons of the tolerable location offset region.

Algorithm	TLOR	Value of Delay Semi Axis (μs)	Value of Doppler Semi Axis (rad)
Binomial Design Algorithm	$O_{\mathrm{BD}}$	$T_{\mathrm{BD}}=0.72$	$D_{\mathrm{BD}}=0.5783$
Algorithm 1	$O_{1}$	$T_{1}=0.09$	$D_{1}=0.1988$
Algorithm 2	$O_{2}$	$T_{2}=0.09$	$D_{2}=0.1804$
Algorithm 3	$O_{3}$	$T_{3}=0.09$	$D_{3}=0.1804$

**Table 3:** Locations of the simulated targets.

Targets	Delay	Doppler
Target 1	$\tau_{1}=12.4\;\;\;\;\mu \;\;\;s$	$f_{d_{1}}=1.3\;\mathrm{rad}$
Target 2	$\tau_{2}=16.6\;\;\;\;\mu \;\;\;s$	$f_{d_{2}}=-0.7\;\mathrm{rad}$
Target 3	$\tau_{3}=16.6\;\;\;\;\mu \;\;\;s$	$f_{d_{3}}=-1.1\;\mathrm{rad}$
Target 4	$\tau_{4}=20\;\;\;\;\mu \;\;\;s$	$f_{d_{4}}=2.2\;\mathrm{rad}$
Target 5	$\tau_{5}=9.4\;\;\;\;\mu \;\;\;s$	$f_{d_{5}}=-1.8\;\mathrm{rad}$
